# Opium Addiction as a Novel Predictor of Atrial Fibrillation after Cardiac Surgery

**Published:** 2012-09-15

**Authors:** Aria Soleimani, Mohammad Reza Habibi, Farshad Hasanzadeh Kiabi, Amir Emami Zeydi

**Affiliations:** 1 Department of Anesthesiology, Faculty of Paramedicine, Mazandaran University of Medical Sciences, Sari, IR Iran; 2 Department of Anesthesiology, Faculty of Medicine, Mazandaran University of Medical Sciences, Sari, IR Iran; 3 Nursing and Midwifery Department, Mazandaran University of Medical Sciences, Sari, IR Iran

**Keywords:** Opium, Atrial Fibrillation, Thoracic Surgery

*Dear Editor,*

Atrial fibrillation (AF) is one of the most frequent complications after cardiac surgery. It occurs in approximately 20% to 35% of patients after coronary artery bypass graft (CABG) surgery and in more than 50% of patients after valve surgery (1). AF after cardiac surgery is a major cause of patients’ morbidity and mortality. Moreover, it can prolong hospitalization and increase health care costs in these patients (2).

Although many of potential risk factors for development of post-operative AF have previously been demonstrated in cardiac surgery patients (1); the result of a recent retrospective study conducted on 670 patients undergoing CABG showed that opium use is a new predictor of postoperative AF in these patients (3). Opium abuse is a serious health and social problem in many parts of the world (4), and its prevalence among Iranian cardiac surgery patients is between 9% to 15.6% (3,5,6,7). The result of some studies suggested that in patients undergoing CABG, opium addiction is accompanied by increased bleeding following surgery, longer resource utilization and also is a significant predictor of re-hospitalization due to a cardiac disease within six month after surgery (4,6).

Regarding the relatively high prevalence of opium abuse among patients undergoing cardiac surgery and considering its potential role in prediction of AF after cardiac surgery, anesthesiologists and cardiac surgeons should provide better preventive measures when planning surgery in opium addict patients. Since most of the studies performed in this field were retrospective, with inherent limitations, future prospective studies are needed to confirm this association.
